# Usefulness of a mobile application in undergraduate critical care teaching

**DOI:** 10.1186/2197-425X-3-S1-A857

**Published:** 2015-10-01

**Authors:** C Leung, G Joynt, WT Wong, C Gomersall

**Affiliations:** Chinese University of Hong Kong, Anesthesia and Intensive Care, Hong Kong, Hong Kong, China

## Introduction

Very BASIC (VB) is a course for basic assessment and support of critically ill patients that is part of the curriculum for our final year medical school. In 2013, a new digital application for electronic handheld devices was introduced. It was designed to provide quick references and consisted of 57 topics presented in 38 PDF and 2 MP3, 8 flowcharts and 8 medical calculators.

## Objectives

To assess the usefulness of the application in teaching critical care medicine. We hypothesize that students will find it useful and will use it during and beyond the course.

## Methods

Final year medical students were offered access and encouraged to use the application during the course. The login date, time, lesson chosen were collected over 15 months then separate into 3 periods: during the 2 weeks VB course, 34 weeks post-course and 31weeks post-medical school final exam . We used questionnaire to assess students' views on its usefulness (1 = strong disagreement to 5 = strong agreement). The scores of another written test taken at the end of the VB course were compared to that of 2012 students, who did not have access to the application. The application was not to be used during this VB test.

## Results

Of the 160 (96% of class of 166) students that used the applications, 154 (96.3%), 121 (75.6%), and 49 (30.6%) logged in during the VB course, post-course and post-final exam respectively (Table 1). A total of 61531 logs were made. The questionnaire showed that 146 students agreed (score 4 or 5) that the application will be useful after becoming doctors; if include all users, the median (IQR) was 4(4-4.3). Overall, there was tendency to agree with positive statements and to disagree with negative statements about the usefulness of the application (Table 2) . The mean test scores for the VB course of students 2013 compared 2012 were 21.55 ± 3.08 and 22 ± 4.0s, p = 0.024.

**Table 1:** Summary of application logs.

Time period	No. of Students	No. of Logs	Mean No. of logs/student	SD
Overall	160	6531	41	35
During the VB Course	154 (96.3%)	4281	27.8	24.9
post-course to final exam	121 (75.6%)	1851	15.3	18.1
After medical school final exam	49 (30.6%)	399	8.1	11.3

**Table 2:** Student feedback questionnaire part 1

POSITIVE STATEMENTS	No. of replies	Median Score	IQR
It is easy to use the application to find the information that I need	165	4	4-4
The Shock audio guide is a useful concise guide to the initial management	124	4	3-4
The guidelines provide comprehensive coverage of the important factual information required for the immediate care of critically ill patients	161	4	4-4
The emergency epinephrine dosages are easily accessible and the application helped me remember the right dose for different situations	165	4	2-5
The acid-base calculators helped reinforce the method for diagnosing complex acid base guidelines	157	4	3-4
The content of the application as a whole will be useful once I start working as a doctor	164	4	4-4.3

**Table 3:** Student feedback questionnaire part 2.

NEGATIVE STATEMENTS	No. of replies	Median Score	IQR
The application does not run smoothly	165	2	2-3
The GCS calculator does not reinforce the components of the score	160	2	2-3
The Acute respiratory failure audio guide is not a useful concise guide to the initial management	129	2	2-3
There are critical omissions in the guidelines	159	2	2-3
The flowcharts are unlikely to help me manage critically ill patients	164	2	2-2
The content of the application as a whole is not useful in preparing me to work as a doctor	164	2	2-2

## Conclusions

Our results show a high utilization rate of the application amongst students. The number of usage was highest during the course and remained high until the final examination. A third of students continued use it after graduation. Students found the application to be useful as a guide for managing critical illness and predicted continued usefulness beyond graduation. The use of the application did not improve written test performance but also did not lead to complacency as our study shows that many students showed the initiative to use it as a learning tool beyond the course period.

